# Supplementary material for: RNA Sequencing of Medusavirus Suggests Remodeling of the Host Nuclear Environment at an Early Infection Stage
Source: Microbiol Spectr. 2021 Sep 29;9(2):e00064-21. doi: 10.1128/Spectrum.00064-21 (PMC8557863; doi:10.1128/Spectrum.00064-21)
Supplement: SUPPLEMENTAL FILE 2 — Supplemental material. Download SPECTRUM00064-21_Supp_2_seq14.pdf, PDF file, 0.04 MB [file spectrum00064-21_supp_2_seq14.pdf]

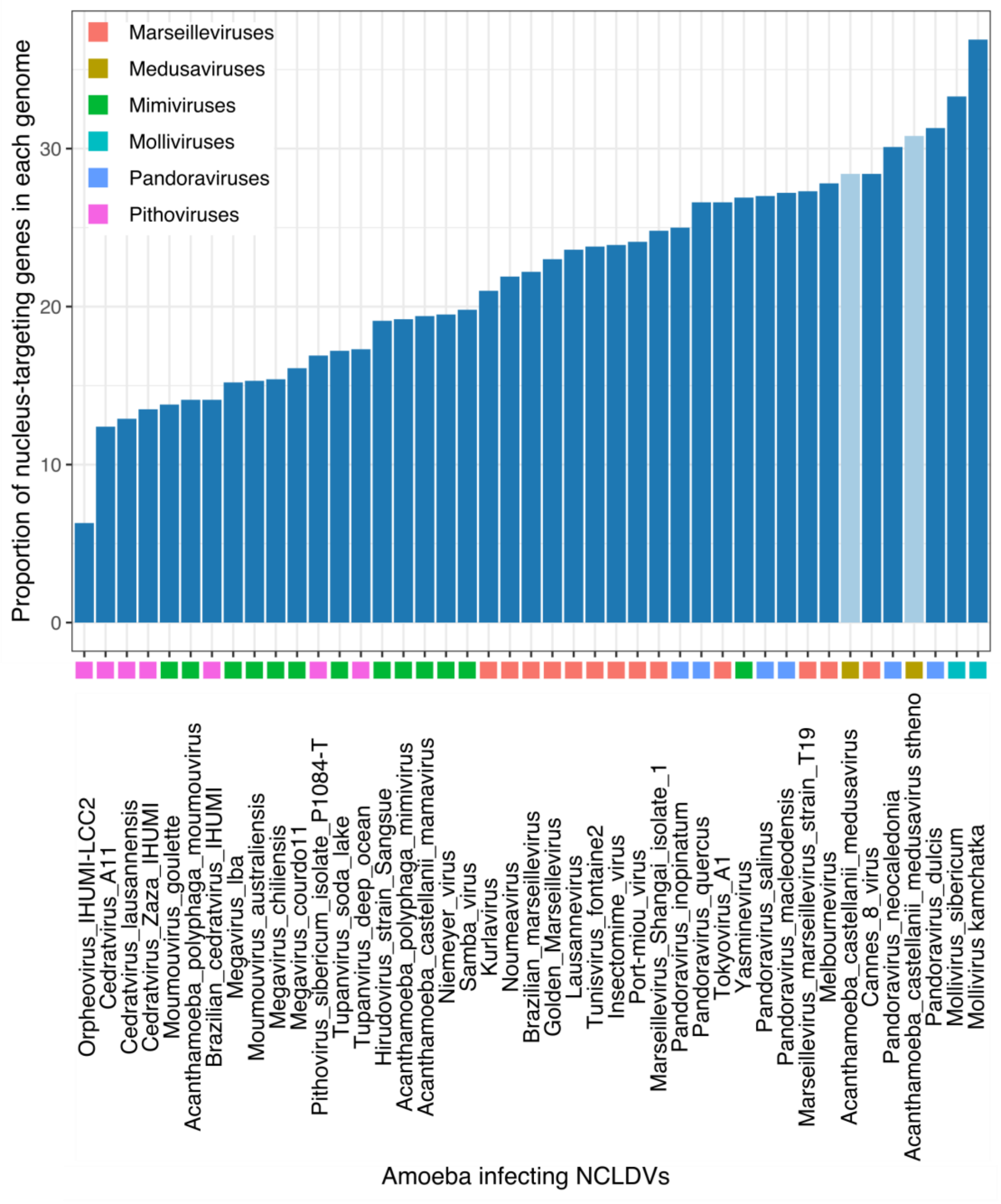

Fig. S1 Deeploc prediction results of other amoeba infecting NCLDVs.

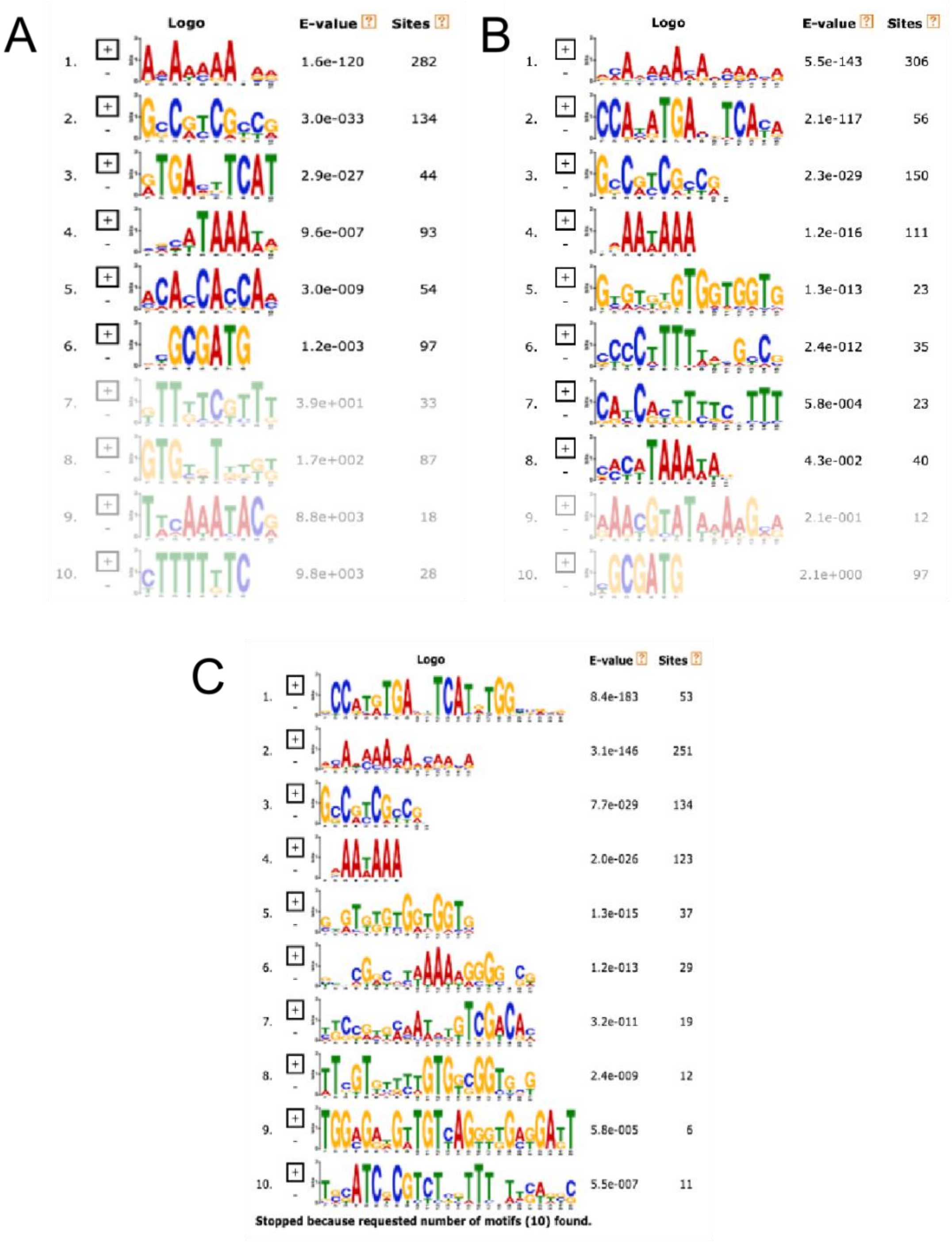

Fig. S2 Promoter motif detected by MEME with motif width ranging from (A) 8-10, (B) 6-15, and (C) 8-25 bp. Motif sequences are ordered according to their e-value.

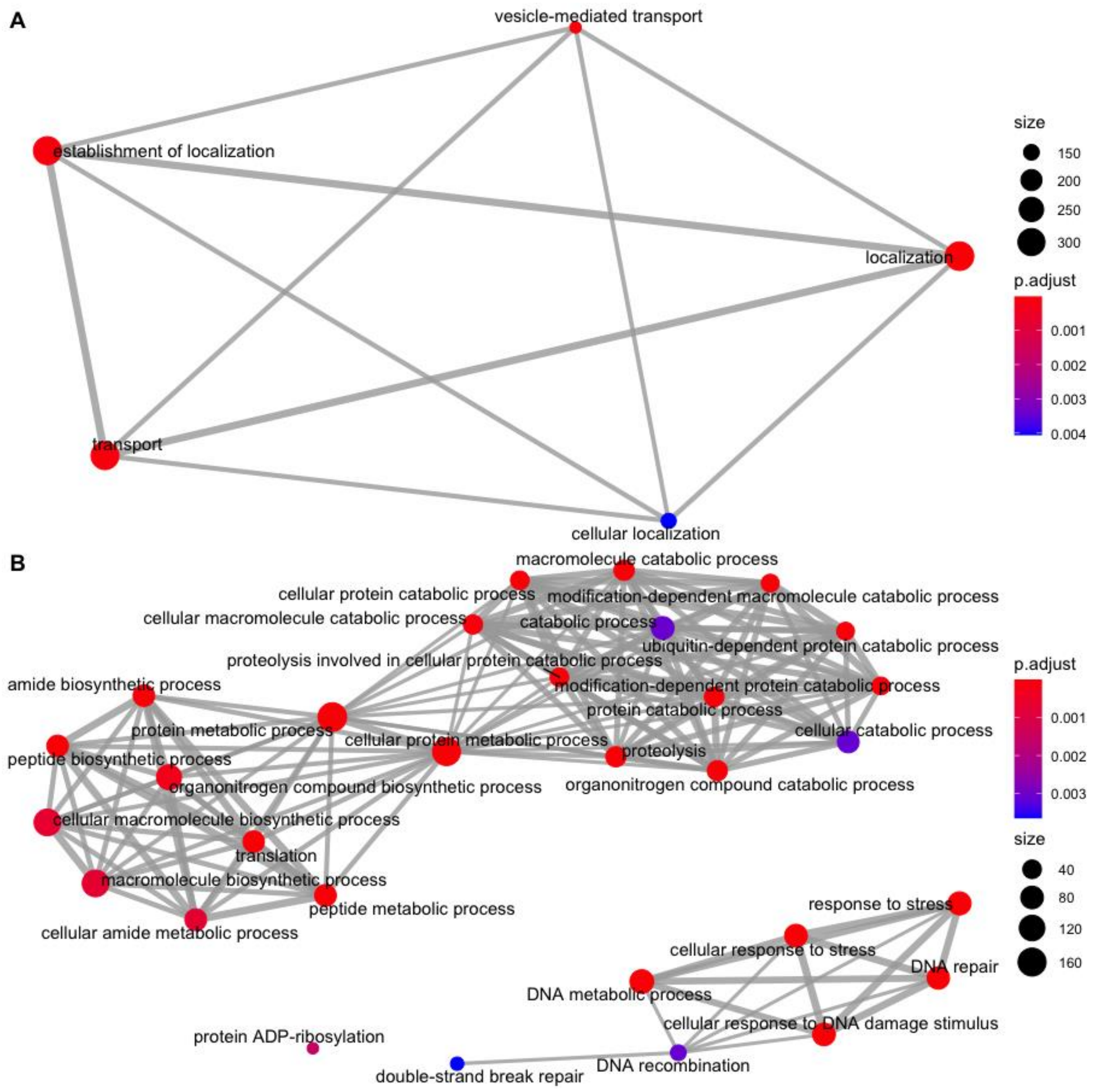

Fig. S3 Enriched map of enriched GO terms. A. GO terms in cluster 1; B. GO terms in cluster 2

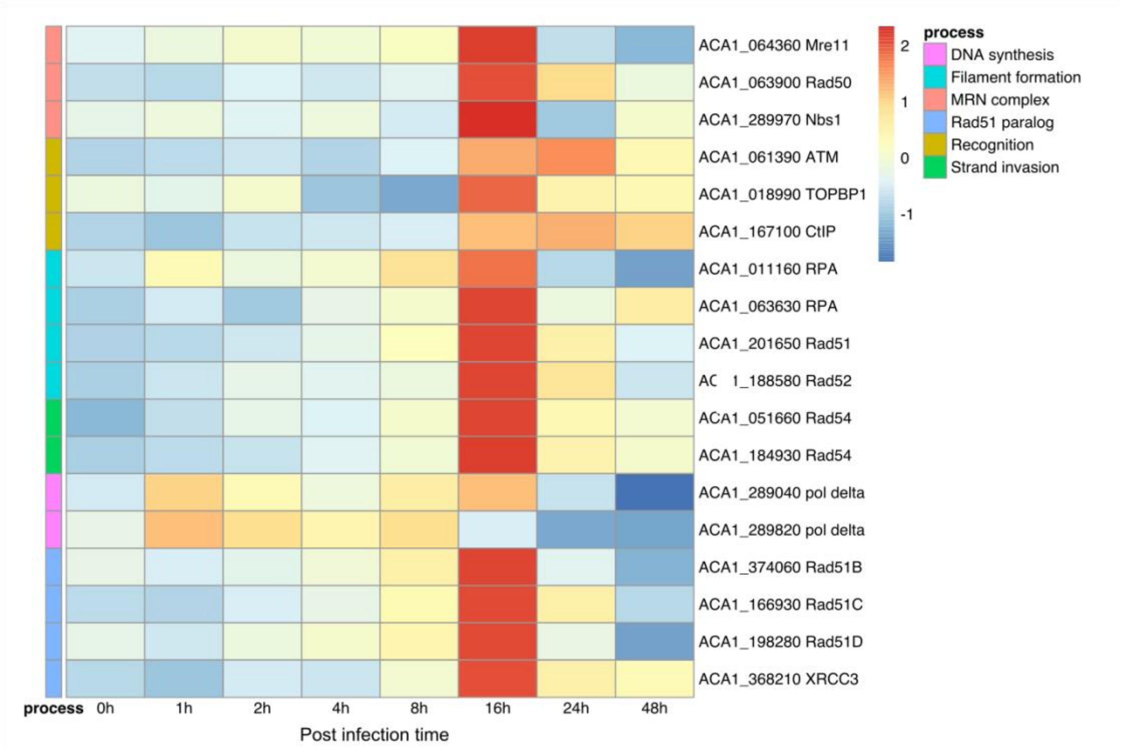

Fig. S4 Expression profile of genes related to homologous recombination repair (KEGG pathway accession number: acan03440). The color scale indicates z-score scaled RPKM values. The process bar on the left indicates the process associated with each gene. Gene names and locus tags are on the right side.

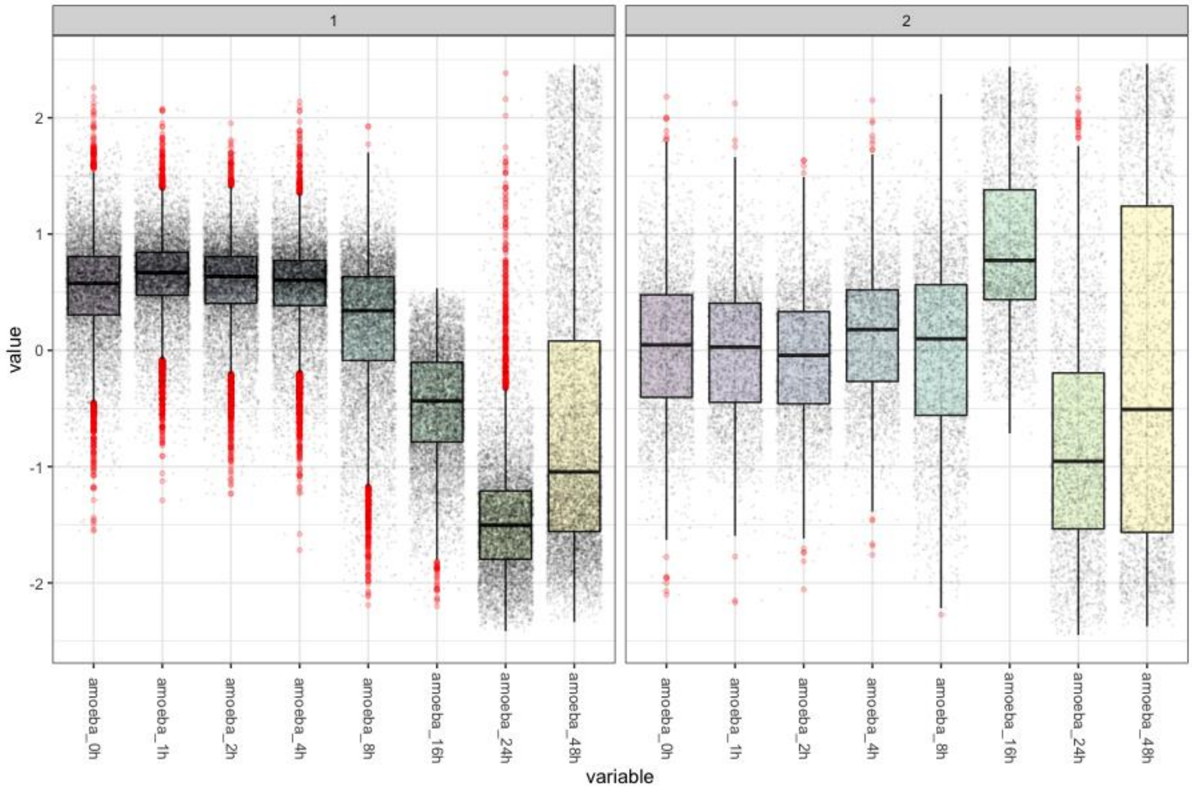

Fig. S5 Expression profile of 2 identified clusters at the end of the experiment.

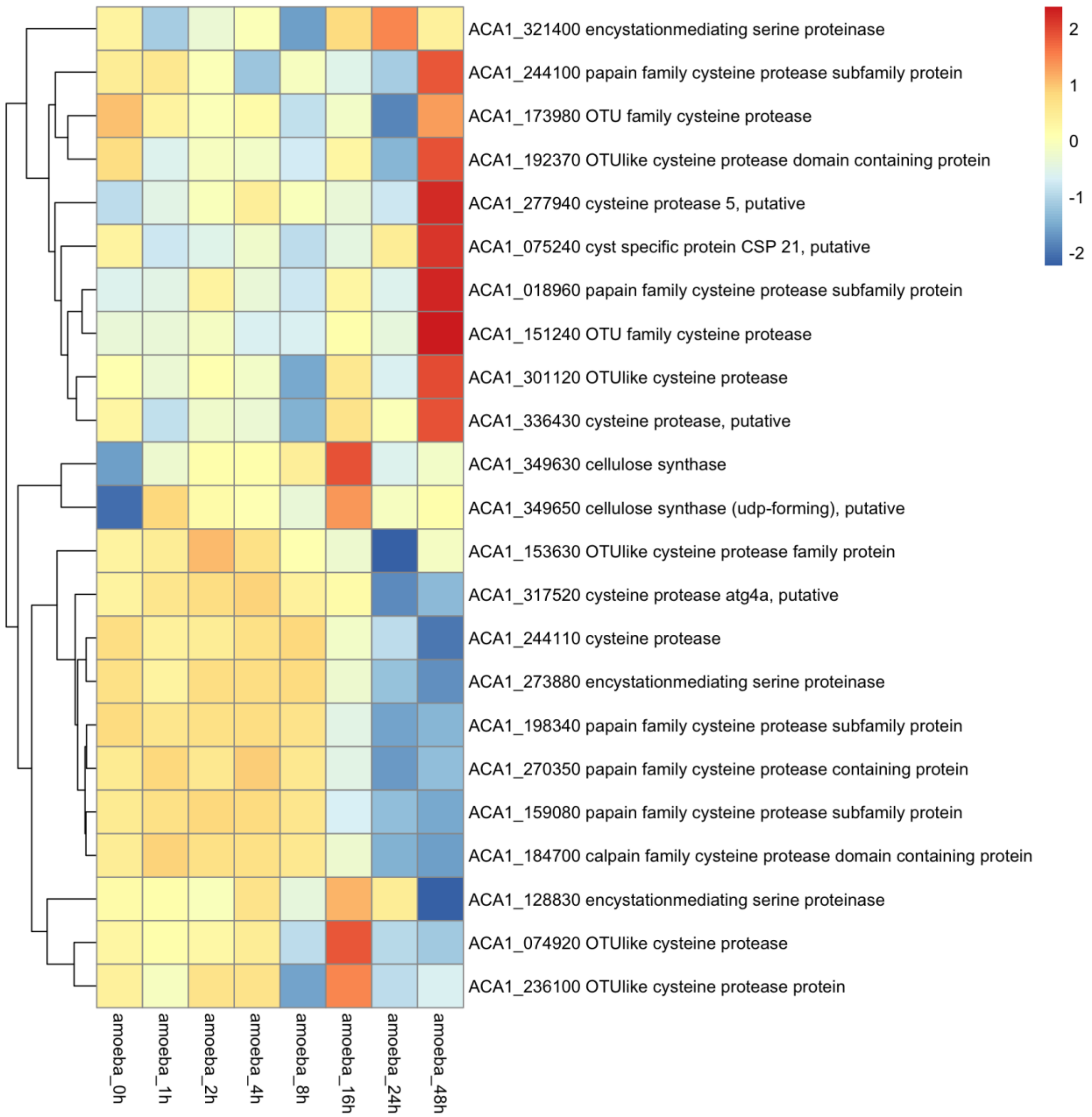

Fig. S6 Expression profile of genes related to encystment (gene name containing cysteine protease, CSP, encystation, or cellulose synthase). The color scale indicates z-score scaled RPKM values. The process bar on the left indicates the process associated with each gene. Gene names and locus tags are on the right side.
