# Supplementary material for: RNA Sequencing of Medusavirus Suggests Remodeling of the Host Nuclear Environment at an Early Infection Stage
Source: Microbiol Spectr. 2021 Sep 29;9(2):e00064-21. doi: 10.1128/Spectrum.00064-21 (PMC8557863; doi:10.1128/Spectrum.00064-21)
Supplement: SUPPLEMENTAL FILE 3 — Supplemental material. Download SPECTRUM00064-21_Supp_3_seq15.pdf, PDF file, 1.0 MB [file spectrum00064-21_supp_3_seq15.pdf]

Table. S1 Motifs found under width parameter: 8-10 nucleotides

Consensus sequence	width	sites	e-value
AMAAAAAVRR	10	282	1.6e-120
GSCRTCGCCG	10	134	3.0e-033
RTGAMTTCAT	10	44	2.9e-027
SVCATAAAWA	10	93	9.6e-007
ACACCACCAM	10	54	3.0e-009
DYGCGATG	8	97	1.2e-003

Table. S2 Motifs found under width parameter: 6-15 nucleotides

Consensus sequence	width	sites	e-value
VMAAMAAMAVMAAMA	15	306	5.5e-143
CCAYATGAMBTCAYA	15	56	2.1e-117
GCCRYCGCCGH	11	150	2.3e-029
DRAAWAAA	8	111	1.2e-016
GYGTKKGTGGTGGTG	15	23	1.3e-013
CCCCTTTTWDHGYCG	15	35	2.4e-012
CAYCRYTTYTCDTTT	15	23	5.8e-004

Table. S3 Motifs found under width parameter: 8-25 nucleotides

Consensus sequence	width	sites	e-value
GCCATRTGAVKTCATRTGGYSRSG	24	53	8.4e-183
VMAAMAAMARMAAMA	15	251	3.1e-146
GCCRYCGYCGH	11	134	7.7e-029

NRAAWAAA	8	123	2.0e-026
GTGTKKGTGGTGGTG	15	37	1.3e-015
BBDCGRCRYWAAAAGGGGNSG	21	29	1.2e-013
YKCCRWKMAAWATGTCGACAC	21	19	3.2e-011
